# Genomic Analysis of 96 *Paenibacillus larvae* Bacteriophages Including 26 from Aotearoa, New Zealand

**DOI:** 10.3390/v17020137

**Published:** 2025-01-21

**Authors:** Danielle N. Kok, Sophia P. Gosselin, Brenham Howard, Steven G. Cresawn, Philippos K. Tsourkas, Heather L. Hendrickson

**Affiliations:** 1 School of Biological Sciences, University of Canterbury, Christchurch 8041, New Zealand; danielle.kok@canterbury.ac.nz; 2 School of Natural Sciences, Massey University, Auckland 0632, New Zealand; 3 Department of Molecular and Cell Biology, University of Connecticut, Storrs, CT 06268-3125, USA; sophia.gosselin@uconn.edu; 4 Biology Department, James Madison University, Harrisonburg, VA 22807, USA; brenhamhoward@gmail.com (B.H.); cresawsg@jmu.edu (S.G.C.); 5 Department of Biostatistics and Medical Informatics, School of Medicine and Public Health, University of Wisconsin, Madison, WI 53706, USA; tsourkas@wisc.edu

**Keywords:** honey bee, American Foulbrood, N-acetylmuramoyl-L-alanine amidase, comparative genomics, Plx1 toxin, host range, putative mobile elements

## Abstract

The bacterium *Paenibacillus larvae* is responsible for the devastating honey bee (*Apis mellifera*) disease American Foulbrood. Research into bacteriophages that infect *P. larvae* is growing rapidly due to increasing antibiotic resistance and restrictions on antibiotic use in beehives in some countries. In this study, we present the sequenced and annotated genomes of 26 novel *P. larvae* phages recently isolated in New Zealand, which brings the total number of sequenced and annotated *P. larvae* phages to 96. The 26 novel phages belong to the pre-existing Vegas or Harrison clusters. We performed a comprehensive genomic analysis of all 96 phage genomes, grouping them into five divergent clusters and two singletons. The majority of these phages are temperate, with the possible exception of three phages that may be lytic. All 96 of these phages encode an N-acteylmuramoyl-L-alanine amidase that serves as their lysin. The amidases are from two divergent clusters, both of which show a high degree of intra-cluster similarity. Six phages and a prophage contain the Plx1 *P. larvae* toxin gene, which we suggest may be mobilizable. This study expands our knowledge of *P. larvae* phages from around the world.

## 1. Introduction

*Paenibacillus larvae* is the causative agent of American Foulbrood (AFB), a destructive and globally distributed disease of the European honey bee (*Apis mellifera*) [1,2]. *P. larvae* is a spore-forming Gram-positive bacterium that is easily spread between hives either via beekeepers or by bees themselves. The spore form of *P. larvae* can persist in the environment for 35+ years [1,3] and is resistant to heat and cold. Together, the ease with which AFB spreads and the hardiness of the spores make this disease particularly devastating to apiculture industries around the world.

Currently, beekeepers take one of two approaches to deal with AFB: treatment with antibiotics, such as Oxytetracycline or Tylosin Tartrate (Tylan^®^ Soluble™) [4], or complete destruction of the infected hive via incineration. Neither approach is ideal; *P. larvae* can become resistant to commonly used antibiotics, and burning hives results in massive financial losses for beekeepers [5,6,7]. In addition, many countries, including the European Union and New Zealand, ban the use of antibiotics in honey production.

An alternative to antibiotics which experienced a resurgence of interest over the last 10 years is the use of bacteriophages (phages) specific to *P. larvae* to either treat the disease or prophylactically protect hives against infection [4,8,9,10]. Phages that infect *P. larvae* were first isolated in the 1950s, but the ease of antibiotic application resulted in little interest in them at the time [11]. With the rise in antibiotic-resistant strains of *P. larvae*, interest in the phages that infect them has grown. The first *P. larvae* phage genome was sequenced in 2013 [12].

Sequencing and annotation of phage genomes is critical for any potential use of phages to treat AFB. It is especially important to identify genes that make the phages lysogenic (such as integrases, Cro/CI, etc.), CRISPR (clustered regularly interspaced short palindromic repeats) protospacers that may neutralise the phages, or genes that would make the phages unsuitable for therapy, such as bacterial toxins and antibiotic-resistance genes [13]. Due to the efforts of several laboratories, including the University of Minho [12,14], the Technical University of Braunschweig [15,16], Brigham Young University [17,18], North Carolina State University [19,20], University of Nevada Las Vegas [21,22,23,24], the Polish Academy of Sciences [25], RTEU University [26], and the University of Canterbury [27,28], 96 unique *P. larvae* phage genomes have been deposited in NCBI GenBank as of this writing. Some of these institutions have not only contributed to the discovery of new *P. larvae* phages, but have also tested the efficacy of phages when treating or protecting honeybee larvae against AFB [4,8,9,10,14]. A comprehensive review of *P. larvae* phage biology [11] and a review of *P. larvae* in therapy applications [29] were published in 2020.

In this study, we first present the genomes of 26 new *P. larvae* phages isolated in New Zealand, representing the first sampling effort that we are aware of in the Southern Hemisphere. Beekeeping of *Apis mellifera* was brought to New Zealand in 1839 and *P. larvae* infection was first noted in 1877 [30,31,32]. The number of incursions of the pathogen since these dates is not known and the importation of live bees and bee products only became strictly regulated with the introduction of the Biosecurity Act 1993 [33]. A recent study of the multi locus sequence types of 164 sequenced New Zealand isolates of *P. larvae* revealed a single dominant sequence type (90.2%) and two minor sequence types [34]. It is not known if this limited diversity is the result of a limited number of introductions or the success of the ongoing eradication efforts. The number of introductions of *P. larvae* phages to New Zealand and their shared ancestry with globally distributed phages has not yet been thoroughly studied.

Previous work undertaken by Stamereilers et al. examined the initial 48 *P. larvae* phages sequenced as of 2018 [24]; the work herein expands on this previous work and includes an additional 48 *P. larvae* phages discovered and sequenced since that time.

Herein, we place the novel New Zealand phages within the context of all 96 extant sequenced *P. larvae* phages and perform a global analysis of *P. larvae* phage genomics. We cluster the phages using percent aligned nucleotide identity (PNI), construct a phylogeny of *P. larvae* phages using total average nucleotide sequence identity (tANI), and perform pairwise genome comparisons to identify conserved an|d divergent genome regions between related phages. We also analyse the phages’ gene product functions, including the large terminase protein, used to infer the phages’ DNA packaging strategy and the presence of genes associated with lysogeny. We also analyse the N-acetyl-L-alanine amidase encoded by all sequenced *P. larvae* phages, which serves as their endolysin, and show that it exists in two highly divergent forms. We examine the prevalence and potential mobility of the Plx1 toxin gene, which confers virulence to *P. larvae*, in some of the global phages and *P. larvae* genomes. This is a crucial addition to the literature as the presence of this toxin renders a phage unsuitable for therapeutic use.

## 2. Materials and Methods

Bacteriophage genomes included in this publication discovered in New Zealand were sequenced and annotated as previously described [27], using the programs Phage Commander [35], DNA Master [36] and a manual curation protocol [37]. Sequenced bacteriophage genomes published by other laboratories were obtained from NCBI GenBank (Supplementary Table S1).

Clustering of *P. larvae* phages was performed as described in previous studies [23,24,38], using PNI and dot plot similarity. For consistency with previous studies, PNI between whole genomes was calculated by constructing a multiple alignment using ClustalW [39] in Geneious (Geneious 10.2.2, Auckland, New Zealand, (https://www.geneious.com) (accessed on 9 December 2024)) with the default settings (IUB cost matrix, gap open cost = 15, gap extend cost = 6.66). Phages were clustered together if their PNI was greater than or equal to 60%, and phages were included in a sub-cluster if their PNI was greater than or equal to 90%, as in previous work [23,24]. Singletons were identified as having less than 60 PNI to any other phage. Dot plots were created using Gepard 2.1 (Cube, Vienna, Austria (https://cube.univie.ac.at/gepard) (accessed on 9 December 2024) [40]. Genome maps were made using Phamerator 2024 (James Madison University, Harrisonburg, VA, USA), a tool for comparative phage genomics (https://phamerator.org) (accessed on 9 December 2024). Phamerator uses the “Align Two Sequences” program contained within BLAST and sets the BLAST E-value threshold at 1 × 10^−4^.

A phylogenetic network of phage genomes was created using total Average Nucleotide Identity (tANI) as the distance metric. tANI is a whole genome distance approach that calculates evolutionary distances by using a modified ANI method [41]. Pairwise BLAST alignments are carried out on all genome pairs in the dataset, after which results are filtered according to percent identity and coverage cutoffs before being used to calculate the average nucleotide identity and alignment fraction between genome pairs. tANI itself is the negative logarithm of the product of the average nucleotide identity times the alignment fraction. The code used as well as additional details can be found here at https://github.com/sophiagosselin/tANI_tool (accessed on 9 December 2024). The tANI distance matrix was imported into SplitsTree4 version 4.18.2 (University of Tuebingen, Tuebingen, Germany) [42]. The network was constructed using a NeighbourNet transformation, ordinary least squares variance, and a lambda fraction of 1 [43]. Gene-level amino acid sequence alignments were constructed using MAFFT [44,45] with default settings in Geneious 9.0.5 (auto algorithm, scoring matrix of 200PAM/k = 2, and preserving original sequence order).

Repetitive elements were found in genomes containing a toxin using the Find Repeats tool in Geneious (Geneious 9.0.5, Auckland, New Zealand, (https://www.geneious.com) (accessed on 9 December 2024)), with a minimum repeat length set to 20 bp and maximum mismatches set to 25%. Hairpins were visualised using VectorBuilder 2024 (VectorBuilder, Chicago, IL, USA (https://en.vectorbuilder.com) (accessed on 9 December 2024)). Trees were made by aligning the genomes using MAFFT and then using the default Geneious Tree Builder Neighbor joining. Bacterial genomes included in this publication discovered in New Zealand were sequenced and annotated as described elsewhere [28]. Bacterial genomes published by other laboratories were obtained from NCBI GenBank (Supplementary Table S2).

## 3. Results

### 3.1. Phage Geographical Locations, Sources and Life Cycle

**Figure 1:**
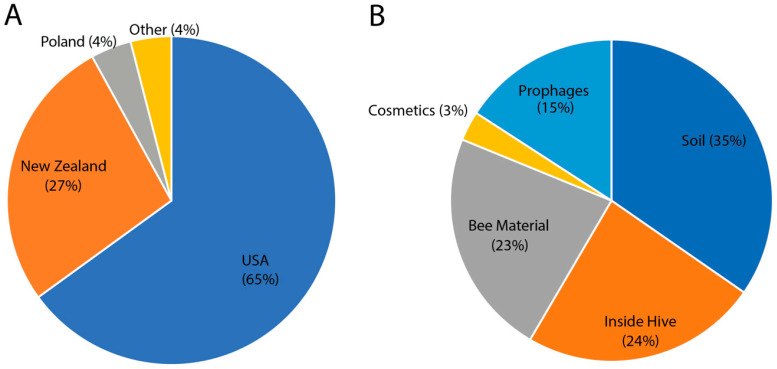
Distribution of geographic locations and sources of 96 *P. larvae* phages. (**A**) The geographic locations of the 96 *P. larvae* phages. (**B**) Sources the *P. larvae* phages were isolated from.

A total of 96 *P. larvae* phages have been isolated, sequenced and annotated to date (Supplementary Table S1). We have contributed 26 to this collection (Table 1). Of the 96, 65% are from the United States (Figure 1A), predominantly three institutes (Brigham Young University, North Carolina State University, and the University of Nevada, Las Vegas), and 27% are from New Zealand. The remaining 8% were discovered in Germany, Poland, Portugal, and Turkey (Figure 1A). Phages have been isolated from several different sources (Figure 1B), with 35% isolated from soil samples collected around beehives and 24% isolated from inside the beehive, including frames and combs. Bee debris (dead bee material) accounted for 23% of the samples, and interestingly 3% came from cosmetics containing bee products. The remaining 15% were prophages isolated from various *P. larvae* strains, having been induced or released from a strain.

### 3.2. New Zealand P. larvae Phages

The 26 new *P. larvae* phage genomes isolated from New Zealand have been previously described [28]. Briefly, these phages are between 40 kbp and 44 kbp in length with 70–83 genes (Table 1). The packaging strategy used by all 26 New Zealand phages is the 3′ cohesive end strategy, as previously described [24]. All 26 NZ phages were lytic in vitro, but their genomes contain an integrase, indicating that they are capable of a temperate lifestyle and have the potential to be lysogenic.

### 3.3. Clustering of New Zealand P. larvae Phages

#### 3.3.1. Clustering Using Percent Nucleotide Identity

**Figure 2:**
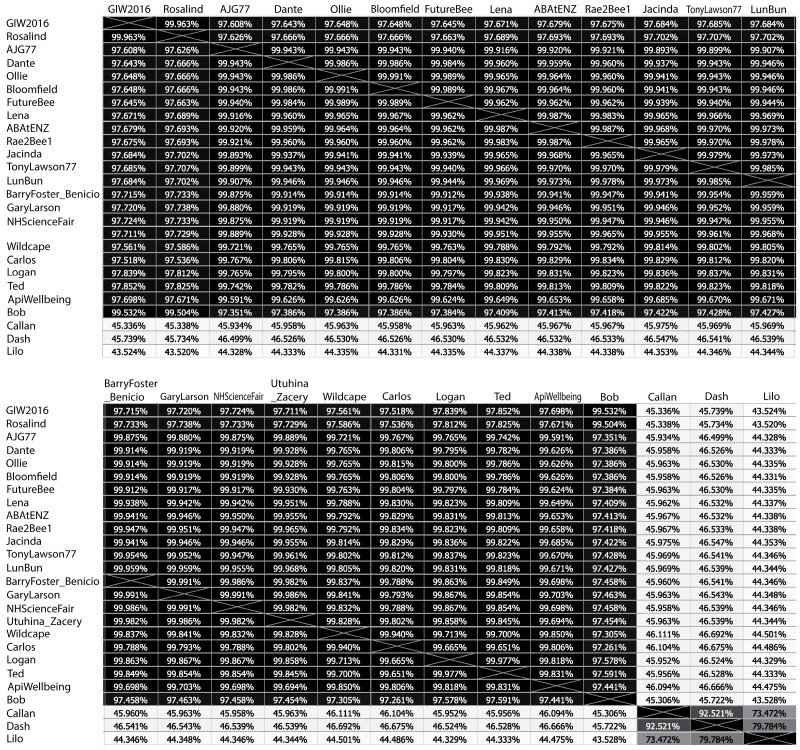
Clustering of New Zealand *P. larvae* phages based on percent nucleotide identity (PNI).

Clustering of the New Zealand *P. larvae* phages was based on percent nucleotide identity (PNI) (Figure 2). Using a 60% PNI clustering cutoff, and 90% for belonging to a subcluster [23,24], we found evidence for two distinct clusters of phages in New Zealand. Callan, Dash, and Lilo form one cluster. Callan and Dash form a sub-cluster, while Lilo forms a separate sub-cluster due to relatively low PNI values with Callan and Dash at 73.49% and 79.81%, respectively. The remaining 23 phages form a separate cluster. Phages in this second cluster have a very high degree of similarity, ranging between 97.3% and 99.9% (Figure 2). A PNI similarity of more than 99.9% indicates that there are approximately 40 base pairs that differ between the two phages based on a ~40 kb genome.

In Stamereilers et al. [24], a PNI cutoff of 99.975 was used to determine whether to publish two phages as distinct or consider them identical. New Zealand phage pairs with PNI greater than 99.975% are usually identical at the amino acid level; however, four groups of phages (described below) have at least one amino acid difference between them. ABAtENZ, Rae2Bee1, Lena, LunBun, TonyLawson77, and Jacinda have a mutual PNI between 99.965 and 99.987%. BarryFoster_Benicio, GaryLarson, UtuhinaGold_Zacery, and NHScienceFair have PNI between 99.982% and 99.991%. Bloomfield, Ollie, FutureBee, and Dante have PNI between 99.984% and 99.991%. Lastly, Logan and Ted have a PNI of 99.977%. Given this large number of exceptions, we propose to replace the 99.975% PNI cutoff with amino acid identity as the criterion for whether to consider two phages identical.

#### 3.3.2. Clustering Using Dot Plot Analyses

**Figure 3:**
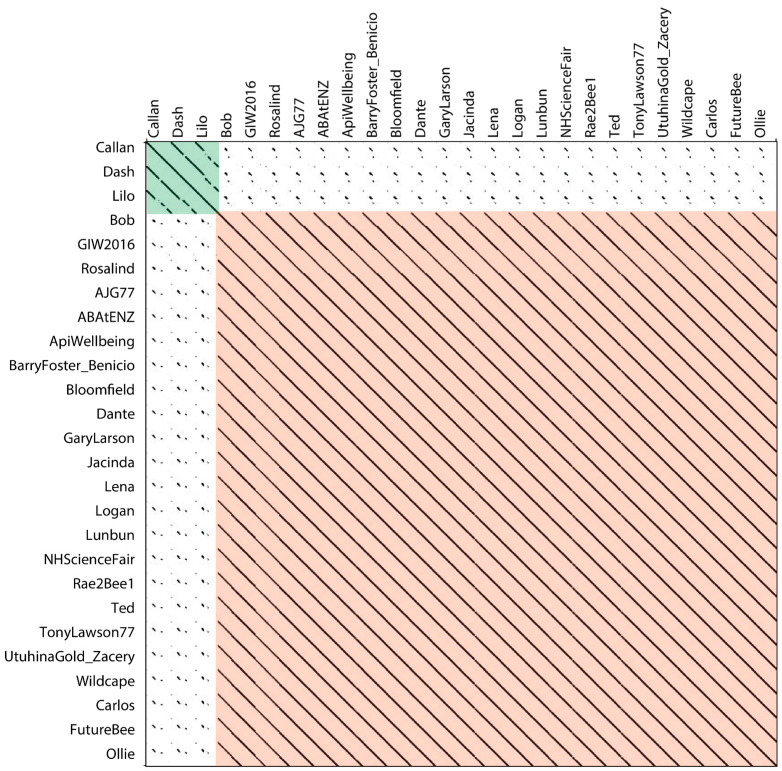
Dot plot of 26 New Zealand *P. larvae* phages displayed using Gepard [40]. Phages grouped into two clusters. The cluster highlighted in red contains 23 phages with a high degree of similarity. The cluster highlighted in green contains three phages with Callan and Dash showing a high degree of similarity and Lilo showing slightly less similarity.

We compared clustering results obtained using PNI values with clustering performed by visual clustering via dot plots. Dot plots allow one to compare the degree of similarity between sequences while providing an indication of synteny. Our approach followed that of Hatfull et al. [38] in which two phages are placed in a cluster if they show evident sequence similarity over at least 50% of the length of the smaller two genomes. Dot plot analyses were performed pair-wise for all our phage genomes using Gepard [40]. The results supported two distinct visual clusters in our New Zealand phages (Figure 3), in agreement with the clustering determined by PNI (Figure 2). The same 23 phages form a single large cluster (red box in Figure 3), while the smaller cluster contains Callan, Dash, and Lilo (green box in Figure 3). Two regions at the beginning of the genomes appear to be highly conserved in all the New Zealand phages.

#### 3.3.3. Pairwise Genome Map Comparisons NZ Phages with Phamerator

**Figure 4:**
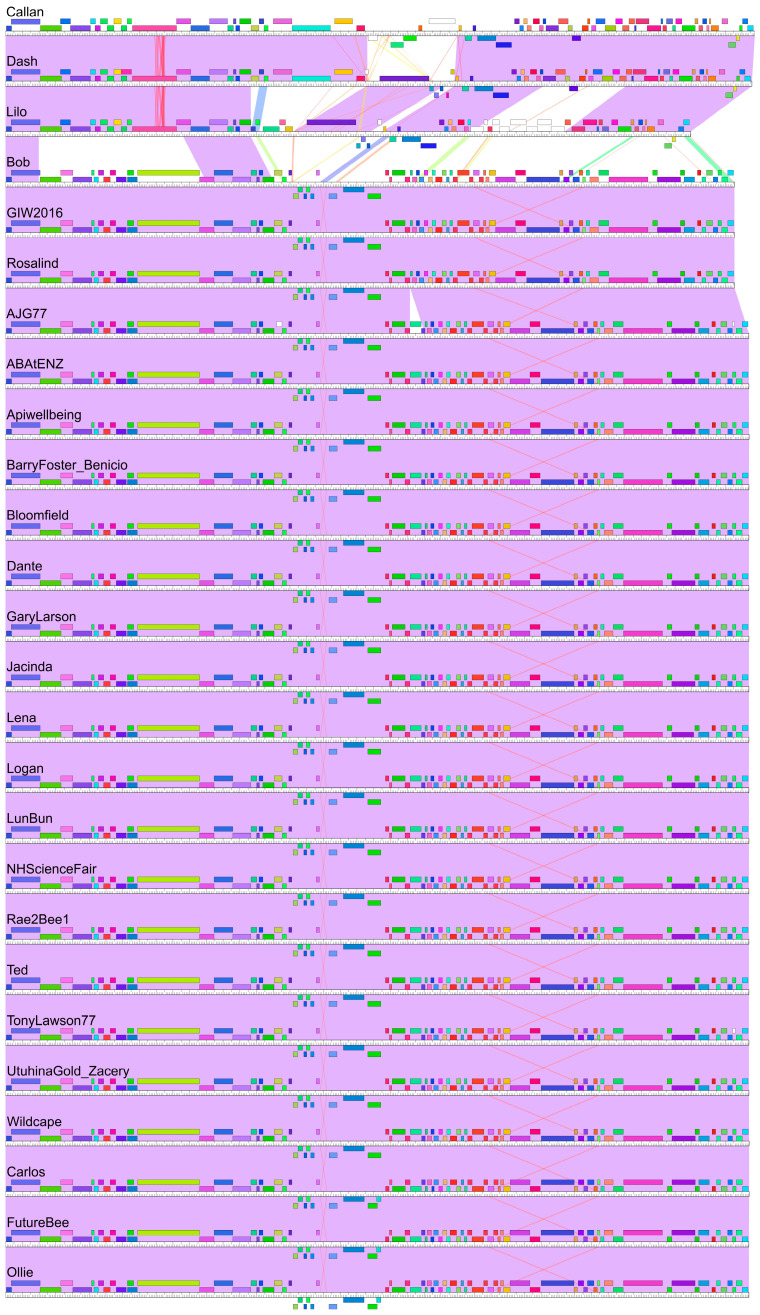
Two clusters of New Zealand *P. larvae* phages. Genome maps of 26 New Zealand *P. larvae* phages created with Phamerator. Coloured boxes represent genes, which are coloured according to their pham assignments. Shading between the genomes indicates how similar an aligned region is at the nucleotide level according to the E-value, with purple depicting an E-value of zero, white indicating no significant similarity, and red indicating similarity at the cut-off threshold of 1 × 10^−4^.

Genome maps were constructed with a Phamerator for the purposes of identifying conserved and divergent regions between phages (Figure 4). Phages Callan and Dash show a high degree of sequence conservation across the majority of their genomes. The most significant synteny break is in the middle of these genomes and contains five genes in Callan and three in Dash. In both Dash and Lilo, this region contains a *P. larvae* toxin, Plx1 (investigated further below), which contributes to pathogen virulence (large dark purple gene in Figure 4), but which is absent in Callan. Phage Lilo also has two other regions that differ from phages Callan and Dash.

Two genomic regions are conserved between the two major New Zealand phage clusters. These broadly conserved regions include the small and large terminases at the front of the genome and a region containing tail proteins, a holin and an N-acetylmuramoyl-L-alanine amidase. Within the larger cluster of 23 phages, Bob, GIW2016 and Rosalind are different from the other phages due to the deletion of two genes in the middle of their genomes that are not present in other phages (Figure 4).

### 3.4. Clustering of All 96 P. larvae Phages

We next clustered the 26 New Zealand *P. larvae* phages with the 70 *P. larvae* phage genomes available on NCBI GenBank using PNI and performed pairwise genome map comparisons with Phamerator. As before, 60% PNI was used as the criterion for inclusion in a cluster, and 90% PNI for inclusion in a subcluster. As a metric of comparison, two randomly generated 40 kb sequences aligned with ClustalW have 40% PNI between them [24].

#### 3.4.1. Global *P. larvae* Phage Clustering Using PNI and Total ANI

**Figure 5:**
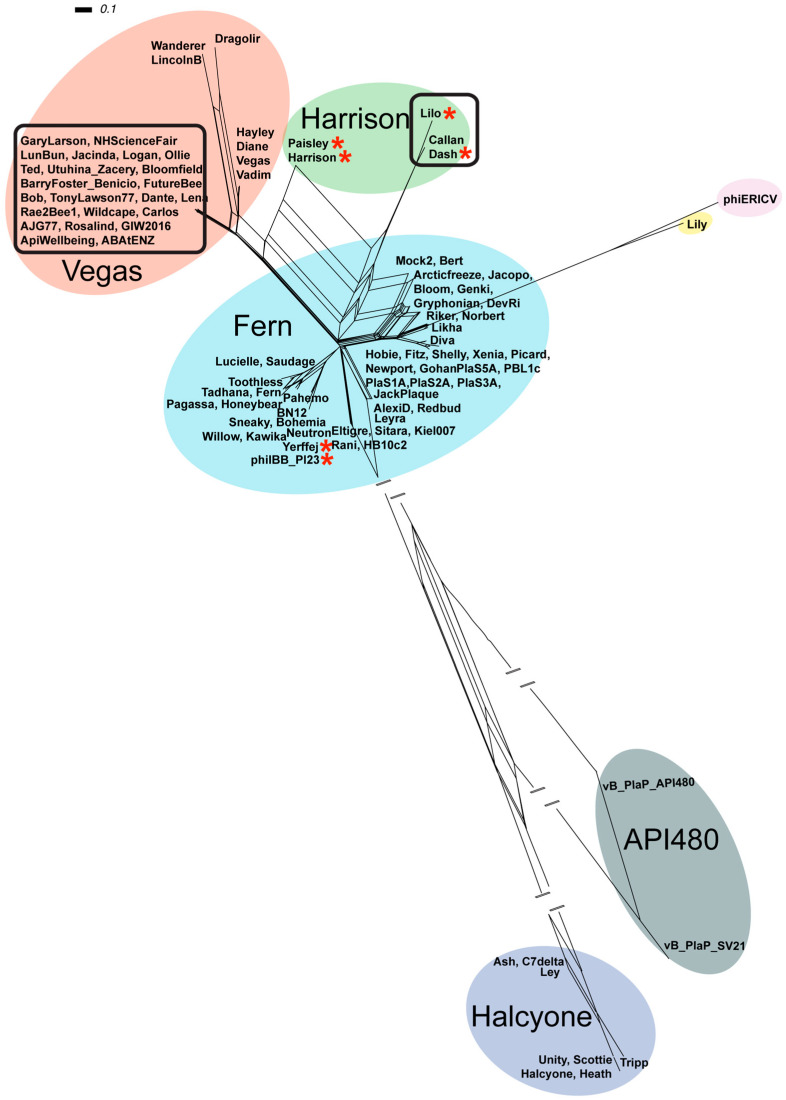
Phylogenetic network of 96 *P. larvae* phage genomes using NeighborNet transformed tANI distances, visualised and constructed via SplitsTree. Coloured circles indicate phages grouped into a cluster. Blue = Fern cluster, Red = Vegas cluster, Purple = Halcyone cluster, Green = Harrison cluster, Yellow for singleton Lily, Pink for singleton phiERICV and Grey for the API480 cluster. Asterisks indicate phages that contain the Plx1 toxin, see text for details. New Zealand *P. larvae* phages are inside black boxes. Dashes (//) indicate shortened branches for the sake of visualisation.

The 96 sequenced *P. larvae* phage genomes were clustered by PNI (Supplementary Table S3), and total ANI, which was then used to generate a SplitsTree (Figure 5). With both methods, the 96 *P. larvae* phages fall into five clusters and two singletons. Four of the five clusters have previously been discovered and have been named after a “representative” phage from each cluster (Fern, Harrison, Vegas, Halcyone) [24]. The new cluster consists of vB_PlaP_API480 and vB_PlaP_SV21, while Lily and phiERICV are singletons, the latter being new since the work undertaken by Stamereilers et al. in 2018 [24].

The Fern cluster (blue in Figure 5) is the largest cluster and contains 49 phages in 16 subclusters, including 2 subclusters that contain more than 10 phages, and 7 subclusters that contain only 1 phage (Supplementary Table S3). This cluster thus contains slightly over half of sequenced *P. larvae* phages. The Fern cluster does not contain any New Zealand *P. larvae* phages. With the exception of vB_PlaP_API480, vB_PlaP_SV21 and phiERICV, all non-New Zealand phages discovered since 2018 (19 in total) are in this cluster. We analysed the PNI of all 96 phages for sets of distinct phages with PNI greater than 99.975%. Two such sets of phages were noted, both in the Fern cluster: Newport and Fitz, isolated in North Carolina in 2014 and 2016, respectively, have PNI of 100%, indicating that they are the same phage; and Riker (a prophage) and Norbert have PNI of 99.990%, but with a single amino acid difference.

The next largest cluster is the Vegas cluster (red in Figure 5), which now contains 30 phages (up from seven previously) within four sub-clusters. The group of 23 highly similar New Zealand phages forms its own subcluster within the Vegas cluster, with PNI ranging from 71 to 76% with phages Vegas, Vadim, Diane, and Hayley, and from 52 to 60% with phages Wanderer, LincolnB, and Dragolir (Supplementary Table S3). The group of 23 New Zealand phages is the largest extant *P. larvae* phage subcluster. Dragolir, Wanderer, and LincolnB are somewhat distant from the other phages in the cluster and are only part of the cluster due to Dragolir having 61% PNI with Diane/Vadim/Vegas and LincolnB having just 60% PNI with NZ phage Bob, as determined by pairwise alignment (Supplementary Table S3). The phages in this cluster are not closely related to those in the Fern cluster, with at most 40% PNI between them.

The Harrison cluster (green in Figure 5) previously contained only two very similar phages, Harrison and Paisley. This analysis joins three novel New Zealand phages (Callan, Dash, Lilo) to this cluster bringing it to five phages in three sub-clusters. The cluster is heterogeneous, as Callan, Dash and Lilo have only 60–68% PNI with Harrison and Paisley (Supplementary Table S3). The phages in this cluster are not closely related to any other phages, with Harrison and Paisley having at most 47% PNI with the 23 NZ phages in the Vegas clusters (Supplementary Table S3).

Phage Lily remains a singleton, having less than 50% PNI with any other *P. larvae* phage, its closest relatives being some phages in the Fern cluster with ~39% PNI and prophage phiERICV at 41% PNI. Prophage phiERICV, isolated from a novel ERIC V *P. larvae* strain in Germany in 2020 [15] is a new singleton. Its closest relative is Lily at 41% PNI, and it is very distant to all other *P. larvae* phages with a maximum of ~31% PNI with the Fern cluster phages.

A new cluster consisting of vB_PlaP_API480 and vB_PlaP_SV21 is now in existence (Supplementary Table S3, Figure 5). Phage vB_PlaP_API480 was isolated by a laboratory in Portugal from a soil sample from Spain [14], while vB_PlaP_SV21 was isolated in Turkey from a prophage [26]. These two phages are highly similar, as their 45 kb genomes have 85% PNI with each other, but are unlike all other *P. larvae* phages, with only 29% PNI with their closest relatives (the NZ phages subcluster in the Vegas cluster). They also have the distinction of being the only two *P. larvae* phages with a *Podovirus* morphology (whereas all others have a *Siphovirus* morphology), while also possessing the longest gene in any *P. larvae* phage genome (~4900 bp). The function of this gene remains unknown.

The Halcyone cluster (purple in Figure 5) remains unchanged as it does not contain any New Zealand *P. larvae* phages or any other new phages since 2018 [24]. The phages in the Halcyone cluster are very distant from all other *P. larvae* phages, with at most ~27% PNI with the phages in the Fern cluster, their closest relatives (Supplementary Table S3).

Thus, 45 of the 48 *P. larvae* phages discovered and sequenced since 2018 fall into previously described clusters, the exceptions being vB_PlaP_API480, vB_PlaP_API480, and the singleton phiERICV. Since the previous comprehensive study [24], three of the four main clusters have been enlarged, there is one new cluster consisting of two phages, and a new singleton.

#### 3.4.2. Pairwise Genome Map Comparisons Global Phages with Phamerator

We also visually examined the 96 *P. larvae* phages via pairwise genome map comparison using Phamerator (Supplementary Figure S1). The first 22 genes of all 49 phages within the Fern cluster are conserved. The thirteen phages in the Pagassa sub-cluster show a high degree of conservation throughout the first half of the genomes and variability throughout the second half. The sub-cluster containing Redbud is made up of genomes that are conserved, with small regions of divergence in the second half of the genomes. The sub-cluster containing Fitz/Newport is largely conserved; Hobie and Picard differ from the other phages in the subcluster by the deletion of a large gene at the end of the genome. The sub-cluster that contains ArcticFreeze is conserved across the whole genome and differs from the Mock2 sub-cluster by a large region towards the end of the genomes. Phages Mock2 and Bert show mutual similarity across the whole genome, as do Norbert and Riker. The remainder of the Fern cluster consist of individual phages, each in their own sub-cluster. The first half of these genomes is conserved, whereas the second half is divergent.

The 23 New Zealand phages in the Vegas cluster have been described previously in this paper. The remaining phages within the Vegas cluster are similar to the 23 New Zealand phages in a region in the second quarter of the genome that is conserved in all phages in the cluster. Phages Diane, Hayley, Vadim and Vegas form a sub-cluster and are highly similar, as are do LincolnB and Wanderer.

The Harrison cluster genomes are conserved at the beginning, end and through a section in the middle of the genome. Dash, Lilo, and Callan, have sequence similarity in the first half of their genomes with the phages in the Fern cluster, whereas Harrison and Paisley, phages discovered in the US, have sequence similarity in the first half of their genomes with the Vegas cluster. Harrison and Paisley have highly similar genomes.

Lily and phiERICV, are singletons, which is clearly shown in the Phamerator maps, with little genome conservation (Supplementary Figure S1) with each other and with other phages. Lily has small areas of genomic similarity with the phages in the Fern sub-cluster and phiERICV. Phages vB_PlaP_API480 and vB_PlaP_SV21 show almost no genome conservation with other phages.

The Halcyone cluster contains eight phages; this cluster of phages is highly divergent from the other *P. larvae* phages, with almost no conservation with other phages, but a high degree of genome conservation within their cluster.

The genome map comparisons show the heterogeneous nature of the *P. larvae* phage genomic landscape. While there is a high degree of conservation within subclusters, there is little genomic conservation between clusters. There is no region that is conserved across all *P. larvae* phage genomes.

### 3.5. P. larvae Phage Gene Product Functions

Given that 45 of the 48 *P. larvae* phages discovered in the past five years, including the 26 NZ phages, fall into previously discovered clusters, the overall picture of *P. larvae* phage gene product functions is broadly similar to 2018 [24]. Approximately 90% of *P. larvae* phage gene products have matches to gene products in public databases, while about half have matches to gene products with assigned functions. Gene products with the following functions have been identified in all sequenced *P. larvae* phage genomes: large terminase, portal protein, major capsid, and N-acetylmuramoyl-L-alanine amidase.

**Figure 6:**
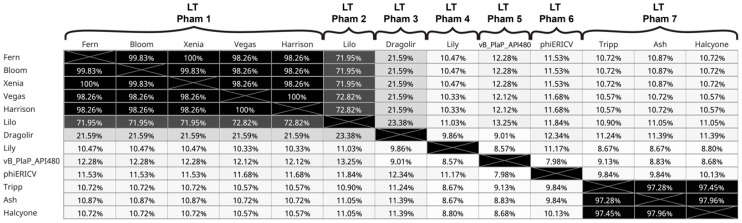
Amino acid identity of the 13 distinct *P. larvae* phage large terminase genes distributed into seven distinct phams. Phages whose large terminase gene is 100% identical to those in the figure are not included.

The large terminase is useful in identifying the phage’s DNA packaging strategy [46]. Based on sequence similarity analysis of the large terminase genes (Figure 6), all *P. larvae* phages whose DNA packaging sequence is known use the cohesive ends packaging mechanism (5′ cohesive ends in the case of Lily, 3′ cohesive ends for all others), except the eight phages of the Halcyone cluster, which use the direct terminal repeats mechanism (DTR). The DNA packaging strategy of phiERICV and the podoviruses vB_PlaP_SV21 and vB_PlaP_API_480 is unknown. Thus, despite the low degree of genomic conservation between clusters, the large terminase and DNA packaging strategy are generally conserved among *P. larvae* phages. Phages from the Fern, Vegas and Harrison cluster have conserved large terminase genes despite low overall inter-cluster genomic similarity, and at least 85 of the 96 sequenced *P. larvae* phages have the same DNA packaging strategy, despite little genomic similarity between them.

**Figure 7:**
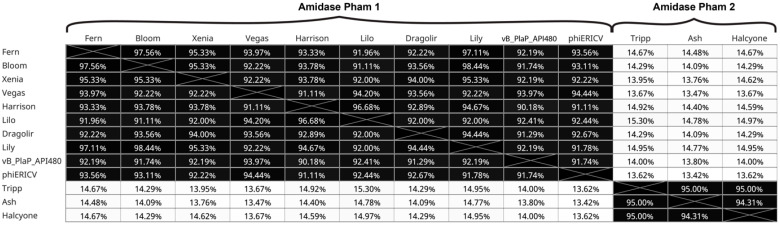
Amino acid identity of 13 distinct *P. larvae* phage N-acteylmuramoyl-L-alanine amidase genes distributed into two distinct phams. Phages whose N-acteylmuramoyl-L-alanine amidase gene is 100% identical to those in the figure are excluded.

All *P. larvae* phages encode a N-acteylmuramoyl-L-alanine amidase that serves as the phages’ endolysin. The *P. larvae* phage endolysin falls into two distinct and highly divergent clusters (Figure 7). Both clusters have high intra-cluster amino acid sequence similarity (>90% amino acid sequence identity AASI) and very little inter-cluster sequence similarity (<15% AASI). The larger of the two clusters includes the amidases of all sequenced *P. larvae* phages except those of the eight Halcyone cluster phages, whose amidases form the smaller of the two amidase clusters. It is important to note that the larger amidase cluster includes the amidase of even highly divergent phages such as Lily, phiERICV, vB_PlaP_SV21 and vB_PlaP_API480, indicating a very high degree of conservation of this protein. The Halcyone cluster amidase genes are 60 aa longer than those of the larger cluster and differ structurally [11]. It is currently not known what the difference is due to, or what effect it might have on lysis, if any. The amidase is flanked by two putative holin genes, which form the lysis cassette. More details on the amidase gene and the lysis cassette have been published previously [11,24].

All but 6 of the 96 sequenced *P. larvae* phages encode gene products indicative of a temperate nature. The *P. larvae* phage integrase/serine recombinase gene is ~1000–1200 bp long, located downstream of the N-acetylmuramoyl-L-alanine amidase gene, on the opposite strand, and flanked by genes with functions related to lysogeny, such as Cro/CI (the lysis/lysogeny switch), excisionase, repressors, and anti-repressors. The lysogeny cassette is downstream of the lysis cassette and upstream of genes related to DNA replication, such as DNA polymerase. A gene encoding integrase has been identified in 69 of the 96 sequenced *P. larvae* phage genomes, while an additional 18 phage genomes encode a large (>1000 bp) gene with functional matches to “serine recombinase” that is almost certainly an integrase. Of the remaining nine phages, Halcyone, Heath, and Scottie encode an identified Cro/CI and a large ~700–1000 bp gene downstream of the amidase and on the opposite strand that likely encodes an integrase. Phages vB_PlaP_API480, vB_PlaP_SV21 and Picard do not appear to encode an obvious candidate for an integrase, but vB_PlaP_API480 was reported as temperate by its discoverers [14], while vB_PlaP_SV21 and Picard were isolated from prophages [26,47]. This leaves phages Diva, Hobie, and Unity as the only *P. larvae* phages that may be purely lytic. These phages’ genomes do not appear to encode anything resembling an integrase, but they do encode genes for antirepressor proteins, suggesting they may be temperate or may have been temperate in the past but subsequently lost this ability.

Six phages encode a *P. larvae* toxin, Plx1. This toxin is of great importance as it confers virulence to *P. larvae* and its presence in the phage genome makes the phage unsuitable for therapy applications. Previously overlooked in the context of *P. larvae* phage genomics, the toxin is discussed in the following section.

### 3.6. Analysis of P. larvae Phage Toxin Plx1

The *P. larvae* toxin gene for Plx1 was detected in four of the five phage genomes in the Harrison cluster: New Zealand phages Dash (GP29), and Lilo (GP26); Harrison (GP30), and Paisley (GP30) from Las Vegas, Nevada; and phages Yerffej (GP26) and phiIBB_Pl23 (GP26) in the Fern cluster [48]. This Plx1 toxin gene, annotated as either ‘toxin’ or ‘RICIN domain-containing protein’, is also found in the genome of *P. larvae* ERIC I (CP019687), within a Harrison cluster prophage (at position 603,427–606,354) [48]. The presence of the Plx1 gene has been shown to contribute to the virulence of *P. larvae* ERIC I, but is not the sole source of its pathogenicity [49].

Plx1 is an AB-toxin, which contains an A-subunit with an ADP-ribosylating domain. This domain is thought to induce apoptosis in eukaryotic cells. The protein also has a B-subunit containing four ricin B-like lectin domains. These help mediate the entry of the toxin into the eukaryotic host cell [49].

**Figure 8:**
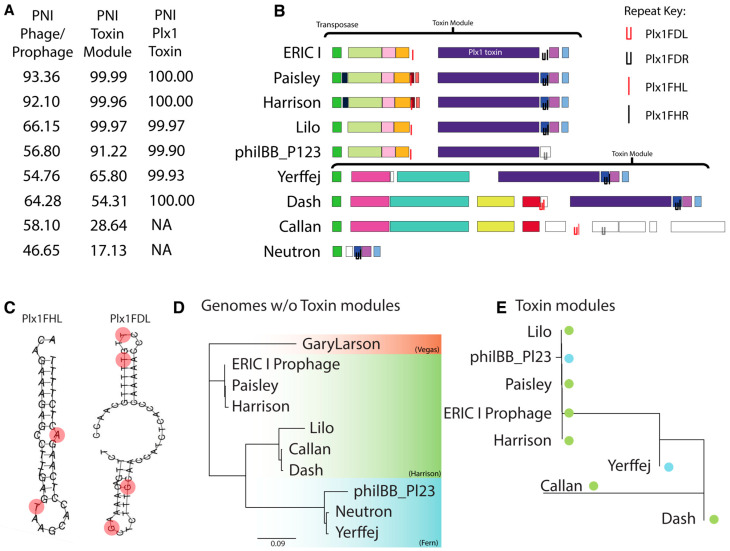
The toxin and its associated module and repeat structure. (**A**) Column one, Percent aligned nucleotide identity (PNI) of each of the phages and prophages listed in B relative to the prophage in *P. larvae* ERIC I (ATCC 9545). Similarly, the corresponding toxin modules (see diagram in (**B**)) in column two and the toxins in column three, all relative to the same in *P. larvae* ERIC 1 (ATCC 9545). (**B**) The genome maps of the putative toxin module. These are simplified genome maps and some genes are on the opposite strand. Gene colours correspond to phamerator maps in Figure 9. See key for the identities of the four repeat sequences (see Supplementary Table S4). Lighter colours indicate less than 100% identity to described repeats. (**C**) Hairpins formed by repeat sequences for Plx1FHL and Plx1FDL made in VectorBuilder (https://en.vectorbuilder.com (accessed on 9 December 2024)). Red circles indicate bases that differ between the left and right repeat sequence. (**D**) Phage Tree based on PNI showing phage genomes with the toxin module removed. GaryLarson is included as an outgroup from the Vegas cluster. (**E**) Gene tree based on the PNI of the toxin module alone. GaryLarson and Neutron are not contained within the toxin module tree as neither contain the module.

Remarkably, this large 975 amino acid toxin is highly conserved in all the phage and prophage genomes in which it is found. Not only is the pairwise amino acid identity between 99.69% and 100% but at the nucleotide identity level, only three nucleotide changes exist across the six instances of this 2928 nucleotide ORF (Figure 8A). For example, we observe 100.00% nucleotide identity in this ORF between the *P. larvae* ERIC I ATCC 9545 from Argentina and New Zealand phage Dash (Figure 8A). This toxin is closely associated in all seven instances with a transposase (Figure 8B green gene) within what we are calling a toxin module. These modules, however, differ dramatically. Three different genetic contents of these modules can be superficially suggested based on the presence or absence of sets of genes (Figure 8B). The ERIC I prophage, Paisley, Harrison, Lilo and PhilBB_P123 have a single toxin module, phage Yerffej has a different set of genes in the module between the transposase and the toxin. Phages Dash and Callan have a shared addition to the Yerffej genes but where Dash has the highly conserved toxin gene, Callan has a separate set of unique genes. Neutron shares the genes that flank the toxin module in the first set but all internal genes, including the toxin, are absent.

This level of nucleotide identity in distantly related genomes suggests that this element may be mobilizing independently of the rest of the region in which it is found (Figure 8A).

**Figure 9:**
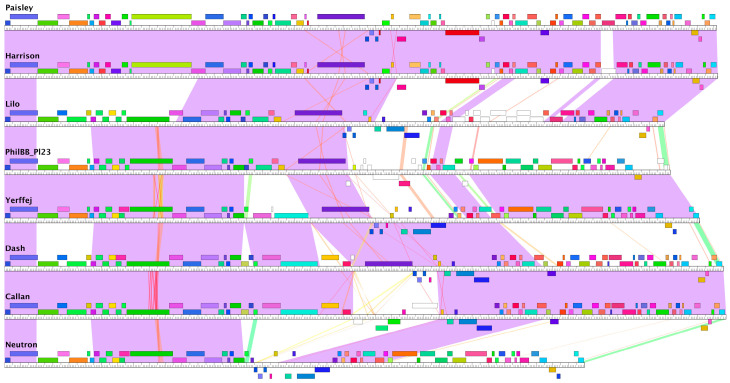
Genome maps of eight *P. larvae* phages displayed using Phamerator. The Plx1 toxin gene is shown as dark purple and located close to the middle of Dash, Lilo, Harrison, Paisley, phiIBB_Pl23, and Yerffej and absent in Neutron and Callan. Colours as described in Figure 4 (https://phamerator.org (accessed on 9 December 2024)).

In addition, inspection of this region reveals a set of repeat regions flanking the toxin gene (Figure 8B, (Key)). These are also seen in the Phamerator map (Figure 9 orange crosses). Two of these attracted our attention, Plx1 Flanking Dash Left/Right (PFDL/R) and Plx1 Flanking Harrison Left/Right (PFHL/R) are named for the first genomic context in which they were recognized. The left and right versions differ slightly as shown. These direct repeats are predicted to form hairpins when single stranded (Figure 8C) and are given in full (Supplementary Table S4).

Phage Callan is highly syntenic with Dash apart from the toxin region and neighbouring genes (Figure 9). Callan contains a 100% match to the repeat sequence Plx1FDL near an incomplete Plx1FHL. A short distance from these repeats, an incomplete and degenerate Plx1FDR is found in a gene that is also present in 10 other Fern cluster phage genomes and the singleton Lily (Figure 8B).

To learn whether these repeats might have previously been recognised in other work, we submitted the complete region between the sets of repeats to the ISFinder database [50]. There was a single significant hit, but this was within the toxin gene and did not include our repeat sequences. Further research will be required to understand the nature of these repeat elements and what role they may play in mobilising the toxin gene.

Together, the phylogenetically heterogeneous appearance of a highly conserved 2000+ nucleotide long open reading frame, flanked by repeat sequences, is curious. The toxin module and the respective phage genomes with the toxin module removed have incongruous phylogenies based on PNI based trees (Figure 8D,E). This observation suggests that the toxin module and its associated transposase is a mobile genetic element that has not previously been described. In addition, the toxin itself has a level of nucleotide identity in these disparate settings that suggests that it is also independently mobilizable.

### 3.7. CRISPR Protospacer Sequences in the 26 New Zealand P. larvae Phages

**Figure 10:**
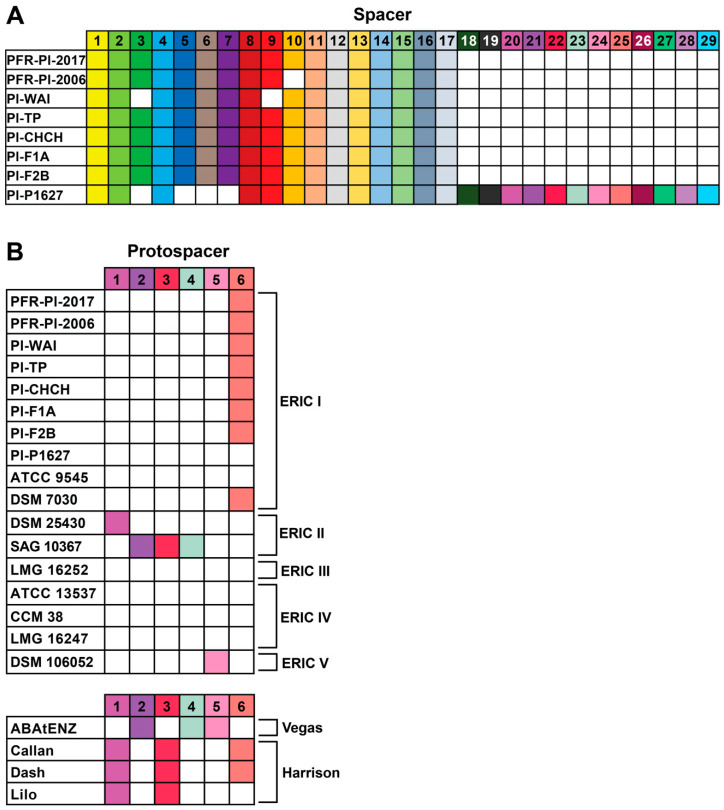
(**A**) Spacers found in eight New Zealand *P. larvae* bacterial strains. (**B**) Protospacers found in 17 *P. larvae* bacterial strains and 26 New Zealand phages. ABAtENZ is used to represent all 23 New Zealand phages within the Vegas sub-cluster.

As previously reported, CRISPR systems were found in the *P. larvae* bacterial strains used to isolate the New Zealand phages [28]. We used CRISPRFinder to detect 29 unique spacers in the eight bacterial genomes that were used to isolate the New Zealand phages (Figure 10A). The sequences of all spacers can be found in Supplemental Table S5. To ascertain if the New Zealand phages contained the 29 spacers identified in our bacterial genomes, we searched their genomes for protospacers using the scan function in DNA Master. We also searched our New Zealand phages for spacers found within nine *P. larvae* strains with complete genomes sequences in NCBI GenBank (Supplementary Table S2). These spacers had previously been identified [51].

Six protospacers were found within the New Zealand phages (Figure 10B and Supplementary Table S6). All 23 New Zealand phages in the Vegas cluster each contained three protospacers, and protospacer 5 was identical to protospacer 9 found in Wanderer and LincolnB [51]. Harrison cluster phages Callan and Dash contained three protospacers each, whilst Lilo only contained two protospacers, but did not contain protospacer 6. The three protospacers in Callan and Dash are identical to those found in Harrison and Paisley and correspond to protospacers 1, 14, and 18 identified previously [51]. Interestingly, protospacer 6, found in Callan and Dash was also found in seven out of eight NZ bacterial strains; these seven isolates are all lysed by Callan and Dash, suggesting the presence of a CRISPR escape mechanism in these phages.

## 4. Discussion

In this paper, we introduced 26 new *P. larvae* phages isolated in New Zealand, and expanded the global analysis of the 48 previously analysed phage genomes for this host by adding a total of 48 new phage genomes annotated since 2018 [24]. The *P. larvae* phages that have been isolated to date have come from North America, Europe, West Asia, and now New Zealand, indicating that *P. larvae* phages are globally spread. It is likely that *P. larvae* phages from other continents will be isolated in the future and added to the 96 described herein.

The New Zealand phages are members of the pre-existing Harrison and Vegas clusters. Three New Zealand phages belong in the Harrison cluster, where they form two sub-clusters. The remaining 23 New Zealand phages are in the Vegas cluster and are highly similar to one another (>90% nucleotide identity), forming a single subcluster.

While the majority of the New Zealand *P. larvae* phages await official species and genus classification, the three Harrison cluster phages described herein have been placed in separate genera. Phage Lilo has been placed in the Harrisonvirus genus whilst phages Callan and Dash have been assigned to the genus Fernvirus [52] (ICTV 2021). Phages Callan, and Dash are at most 41.1 and 41.4 PNI to the Fern cluster phages, respectively (Supp. Table S3). However, these two phages are 59.5 and 65.0 PNI to Phage Harrison, respectively. We would therefore suggest that a re-classification of phages Callan and Dash into the Harrisonvirus genus would be a more appropriate taxonomic assignment.

We have previously described preliminary sequencing of eight New Zealand strains of *P. larvae* [28]. Each of those isolates has either one or two intact prophages that are most similar to either the Harrison or Vegas cluster phages. This suggests that there is a long term association between *P. larvae* in New Zealand and the phages from these two clusters. One is tempted to suggest that these phages arrived in New Zealand as guests in the genome of the pathogen during incursion events and that they have since evolved past the point of self-recognition. Recently, 55 *P. larvae* prophages were discovered in the genomes of 11 isolated *P. larvae* strains from AFB outbreaks around the world [53]. Taken together with the multiple occasions we have seen *P. larvae* phages isolated from lysogens [12,15,17,18,21,26,54], this suggests it is possible the New Zealand *P. larvae* phages could have arisen via excision from the host genome. The existence of a large number of prophages within *P. larvae* genomes is also an important avenue of future research, given that prophages mediate intra and extra-phage resistance.

In addition to the 8 *P. larvae* bacterial isolates mentioned, recent sequencing efforts have added 164 new isolates and identified three distinct MLST sequence types in New Zealand. These different sequence types potentially entered New Zealand during distinct incursions of the pathogen [34]. For at least the past 30 years, the import of bee related products into the country has been extremely restricted [31,33]. The limited number of MLST groups present in New Zealand suggests these restrictions have been effective in limiting additional incursions.

We clustered all 96 sequenced *P. larvae* phage genomes, significantly expanding the previous analysis [24]. The 96 sequenced *P. larvae* phages are grouped into the four main clusters identified in 2018 [24], a new cluster consisting of two phages, and two singletons, one of which is new since 2018. This has expanded the Fern, Harrison and Vegas clusters. The Halcyone cluster is unchanged since 2018 and Lily remains a singleton. The only additions outside of the four main clusters and one singleton are a new cluster consisting of two closely related novel phages with a *Podoviridae* morphology, vB_PlaP_API480 and vB_PlaP_SV21, and a new singleton, phiERICV.

The distribution of *P. larvae* phages thus has not fundamentally changed despite the near doubling of the number of sequenced *P. larvae* phage genomes. This suggests that much of the genomic diversity of these phages may have been discovered, at least within the confines of our current discovery methods. That said, we are far from saturating the genomic space, and few of the phages discovered so far appear to be duplicates of previously discovered phages. The main clusters and singletons are genetically distant from each other, indicating a highly heterogeneous genomic landscape.

In terms of genome length, GC content, and DNA packaging mechanism, most *P. larvae* phages are in the 35–46 kb length range, 40–44% GC content range, and use the “cohesive ends” DNA packaging mechanism. The exception are the Halcyone cluster phages, whose genomes are in the 50–56 kb range, have 48–49% GC content, and use the Direct Terminal Repeat DNA packaging mechanism. The Halcyone cluster phages also have a N-acetylmuramoyl-L-alanine amidase endolysin that is longer by 60 amino acid residues and highly divergent from that of all other *P. larvae* phages. The divergence between the Halcyone cluster phages and all other *P. larvae* phages is the most fundamental observed amongst the *P. larvae* phages and points to an early split in their evolutionary history. Any functional importance of this distinction remains to be characterized, as phages from both main groups are effective at lysing their host.

Roughly half of *P. larvae* phage gene products have similarity matches to genes with known function. *P. larvae* phages encode gene products with structural, assembly, lytic, lysogenic, DNA replication, regulatory, and host-related functions. In this study we focused on the Plx1 *P. larvae* toxin gene that had been overlooked in previous analyses of *P. larvae* phages.

The focal *P. larvae* toxin Plx1 gene homolog was identified in New Zealand phages Dash and Lilo, bringing the total number of phages harbouring this toxin from four to six since 2018. This toxin has been described in the bacterial strain *P. larvae* ATCC 9545 ERIC I [48]. It has previously been suggested that the presence of Plx1 in the *P. larvae* ERIC I genome is a consequence of lysogenic conversion from temperate phages. While the six phages that contain the toxin are temperate, the host or conditions that are permissive to genetic integration of these phages is not currently known. Ebeling and colleagues suggested that Plx1 may be present in distantly related *P. larvae* phages due to an ancestral contribution by the bacterial strain via an abnormal excision event. They further suggest that the presence of the toxin in the prophage region of the bacterial genome is due to lysogenic conversion. Our discovery of a putative mobile element in the proximity of the Plx1 toxin gene suggests that the mobilisation of this toxin may be independent of phage integration. In addition, the nucleotide identity of all instances of this toxin is nearly 100%, which is not consistent with toxin transfer in the context of either the phage or the toxin module in which it is found (Figure 8). Rather, the toxin appears to be a recent arrival in all of these contexts which implies that it is independently mobilizable. This may be an instance of a hitcher element [55]. This potential genetic symbiosis will be the subject of future investigation.

The question of whether these phages can form lysogens with the *P. larvae* host is still an open one. The presence of a potentially highly mobile toxin gene in six *P. larvae* phages with lysogenic potential means that these phages are currently not appropriate for applied use. The presence of this toxin in *P. larvae* phage genomes also highlights the importance of accurate genome sequencing and annotation of any phages with potential for use as treatment agents.

Related to this toxin issue, phage Dash (with an intact toxin gene) was previously used in an in vitro phage cocktail test [28]. The cocktail that included Dash was performing well in these tests. However, upon sequencing and recognising the toxin, we substituted phage Dash with Callan and discovered that while these phages were highly similar outside of the toxin region, this substitution reversed cocktail efficacy in a host dependent fashion [28]. This suggests that phage–phage antagonism may also exist amongst *P. larvae* phages, a finding that will require further investigation.

Comparative analysis was also undertaken on the N-acetylmuramoyl-L-alanine amidase, which *P. larvae* phages use to lyse their host, and which has been found in every *P. larvae* phage genome isolated to date. Previously, 12 distinct N-acetylmuramoyl-L-alanine amidases in 2 highly divergent groups were found, out of a total of 48 sequenced *P. larvae* phage genomes [24]. In this study, we show that there are now 26 distinct N-acetylmuramoyl-L-alanine amidases within the same two highly divergent groups.

The N-acetylmuramoyl-L-alanine amidase is a type of endolysin and is the only *P. larvae* phage protein whose function has been experimentally verified [56]. Endolysins are important as they enable phages to destroy the bacterial cell wall and lead to the rupture of the bacterial host, ultimately resulting in its demise [57,58]. The functional differences between the two different amidase clusters are currently not known, and this would be an interesting future direction.

All New Zealand phages contain CRISPR protospacer sequences found in *P. larvae* genomes. CRISPRs are one of the most easily recognisable and best characterised bacterial defense mechanisms against phages. However, the protospacer containing New Zealand phages were able to lyse their CRISPR sequence-containing hosts, suggesting the existence of an escape mechanism, possibly through anti-CRISPR genes. In order to bring these phages into practical use, the mechanism by which these phages are navigating host defense systems will need to be studied further.

In conclusion, the work contained within this study has updated our knowledge of *P. larvae* phages by clustering all known phages for this host including recently isolated and sequenced genomes from New Zealand and elsewhere. This has enlarged the three main clusters, added cluster API480, and added a new singleton (phage phiERICV). We have analysed the distribution of phage gene product functions with a focus on the large terminase and N-acetylmuramoyl-L-alanine amidase and found that this is largely unchanged since the 2018 analysis. We have also identified a putative mobile element in a set of these phages that contains a toxin that contributes to the virulence of many *P. larvae* strains. In addition, we looked into the CRISPR protospacer sequences in the New Zealand phages and we found that Callan and Dash should be recognised by the CRISPR systems in the *P. larvae* that they infect. This suggests these phages have unrecognized CRISPR escape mechanisms. Finally, we note that there is cause for reassigning phages Callan and Dash to the genus Harrisonvirus, rather than genus Fernvirus.

Some key areas of further study include (1) to ascertain if the toxin and toxin module are mobilizable, (2) to identify the functional differences between the two main classes of amidases, (3) to identify the functions of more *P. larvae* phage proteins including but not limited to CRISPR-escape mechanisms, (4) to characterize, annotate, induce, and test the prophages in *P. larvae* genomes, (5) to identify the mechanisms by which *P. larvae* phages enter and exit lysogeny, and (6) determine whether there are purely lytic *P. larvae* phages.

## Figures and Tables

**Table 1:** *P. larvae* phages isolated in New Zealand. All phage genomes have been reported previously [28].

Phage Name	Genome Length (bp)	No. of Genes	GC Content (%)	Cluster	Accession No.
ABAtENZ	44,419	82	42.97	Vegas	OP503968
AJG77	44,417	82	42.98	Vegas	OP503969
ApiWellbeing	44,429	82	43.01	Vegas	OP503970
BarryFoster_ Benicio	44,421	82	42.98	Vegas	OP503543
Bloomfield	44,419	82	42.98	Vegas	OP503971
Bob	43,553	80	43.03	Vegas	OP503972
Callan	44,768	77	39.69	Harrison	OP503989
Carlos	44,430	83	42.98	Vegas	OP503973
Dante	44,420	82	42.98	Vegas	OP503974
Dash	44,599	79	39.39	Harrison	OP503990
FutureBees	44,417	83	42.98	Vegas	OP503975
GaryLarson	44,420	82	42.98	Vegas	OP503976
GIW2016	43,555	80	43.01	Vegas	OP503977
Jacinda	44,419	82	42.97	Vegas	OP503978
Lena	44,420	82	42.97	Vegas	OP503979
Lilo	40,941	70	40.33	Harrison	OP503991
Logan	44,419	82	42.99	Vegas	OP503980
LunBun	44,421	82	42.97	Vegas	OP494865
NHScienceFair	44,419	82	42.98	Vegas	OP503981
Ollie	44,420	83	42.98	Vegas	OP503982
Rae.2Bee1	44,420	82	42.97	Vegas	OP503983
Rosalind	43,556	80	43.00	Vegas	OP503984
Ted	44,419	82	42.99	Vegas	OP503985
TonyLawson77	44,420	82	42.96	Vegas	OP503986
UtuhinaGold_ Zacery	44,420	82	42.97	Vegas	OP503987
Wildcape	44,430	82	42.98	Vegas	OP503988

## Data Availability

Data available in a publicly accessible repository, for details please see Supplementary Table S1. The original data presented in the study are openly available in NCBI.

## Supplementary Materials

The following supporting information can be downloaded at: https://www.mdpi.com/article/10.3390/v17020137/s1, Figure S1: Genome maps of 96 P. larvae phages displayed using Phamertor; Table S1: 96 P. larvae phages isolated from around the world; Table S2: 17 *P. larvae* bacterial strains; Table S3: Clustering of all 96 *P. larvae* phages based on percent identity; Table S4: Repeat sequences found flanking Plx1 toxin gene; Table S5: Spacer sequences found in eight NZ *P. larvae* bacterial strains; Table S6: Protospacer sequences found in 17 *P. larvae* bacterial strains and 26 New Zealand phages.
