# Supplementary material for: Genomic Analysis of 96 Paenibacillus larvae Bacteriophages Including 26 from Aotearoa, New Zealand
Source: Viruses. 2025 Jan 21;17(2):137. doi: 10.3390/v17020137 (PMC11860570; doi:10.3390/v17020137)
Supplement: Supplementary file 1 [file viruses-17-00137-s001.zip › Supplementary Data 011024.pdf]

Supplementary Data

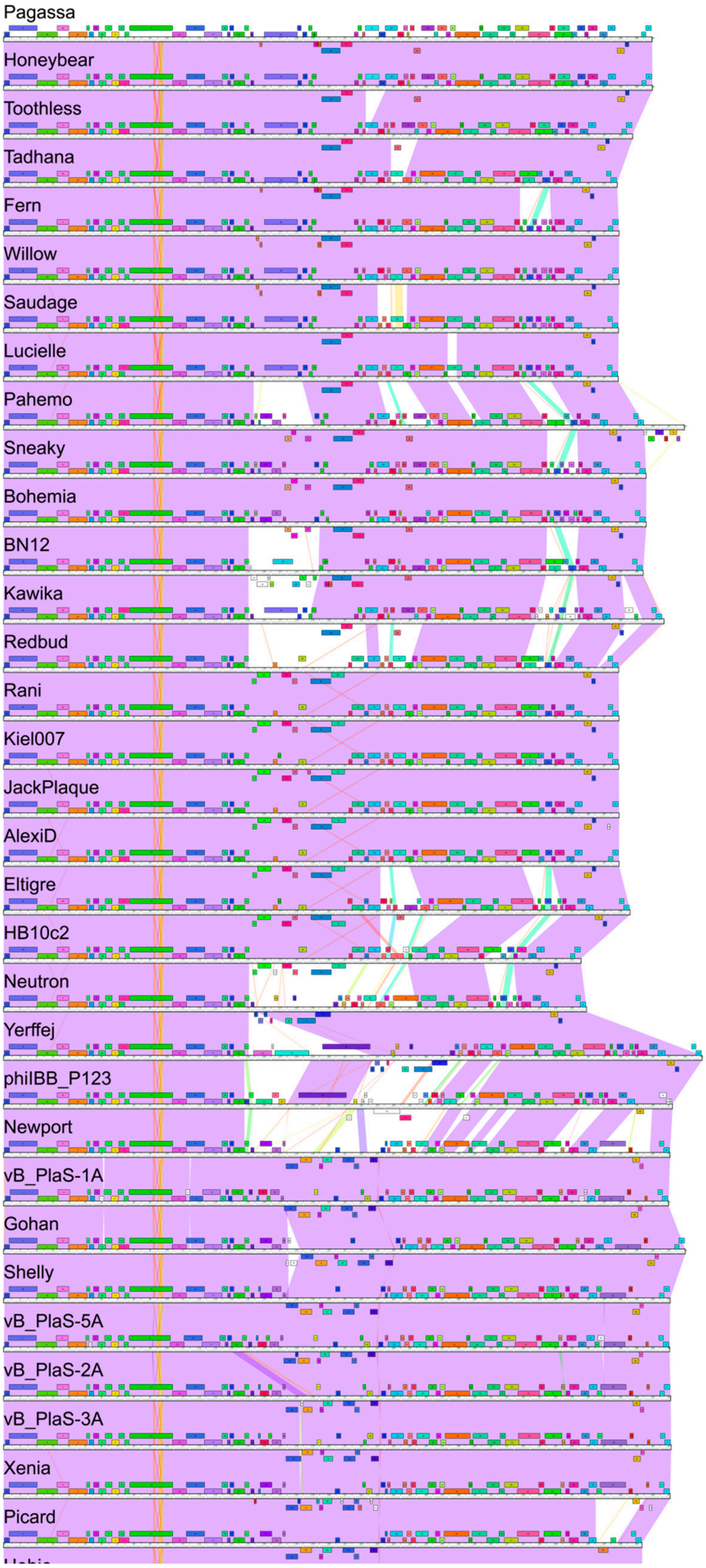

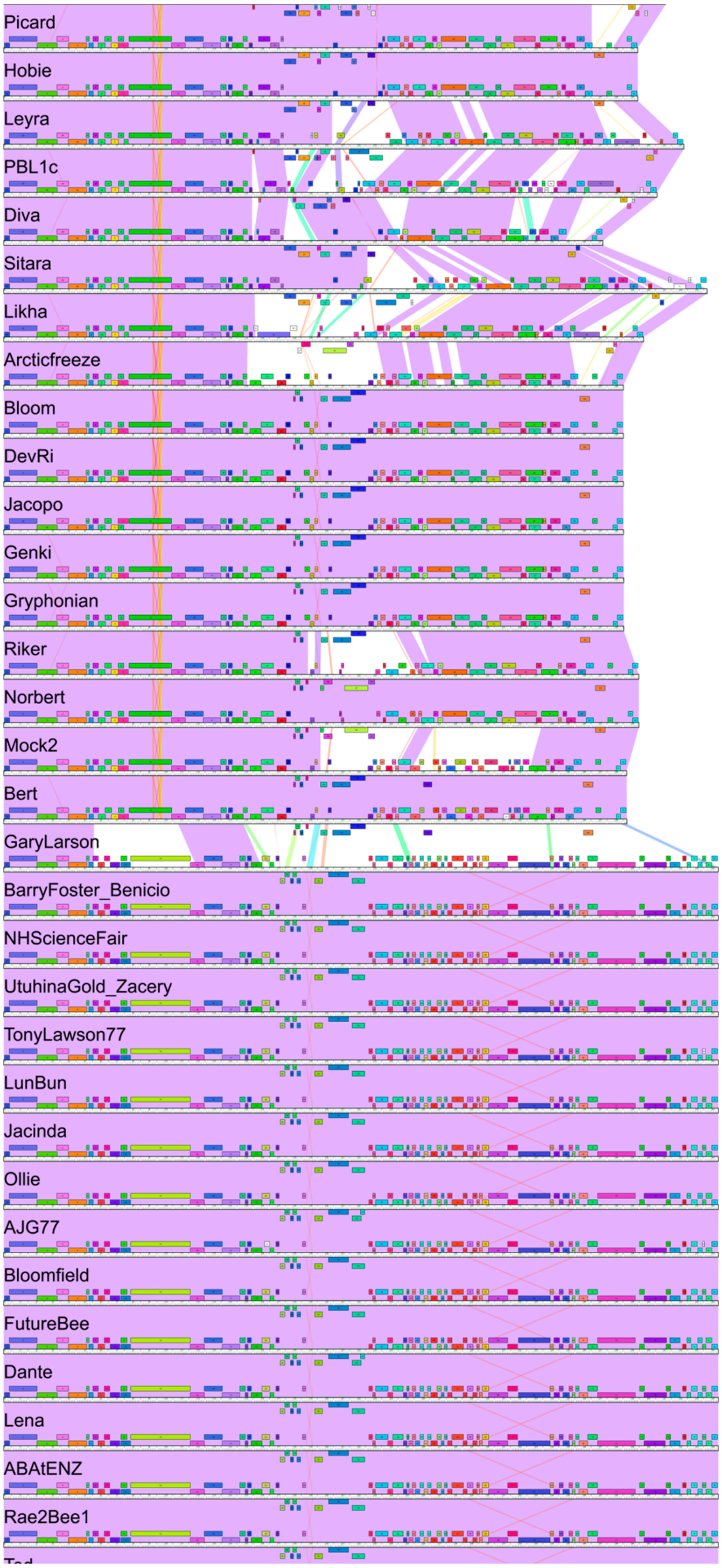
Picard
Hobie
Leyra
PBL1c
Diva
Sitara
Likha
Arcticfreeze
Bloom
DevRi
Jacopo
Genki
Gryphonian
Riker
Norbert
Mock2
Bert
GaryLarson
BarryFoster_Benicio
NHScienceFair
UtuhinaGold_Zacery
TonyLawson77
LunBun
Jacinda
Ollie
AJG77
Bloomfield
FutureBee
Dante
Lena
ABAtENZ
Rae2Bee1

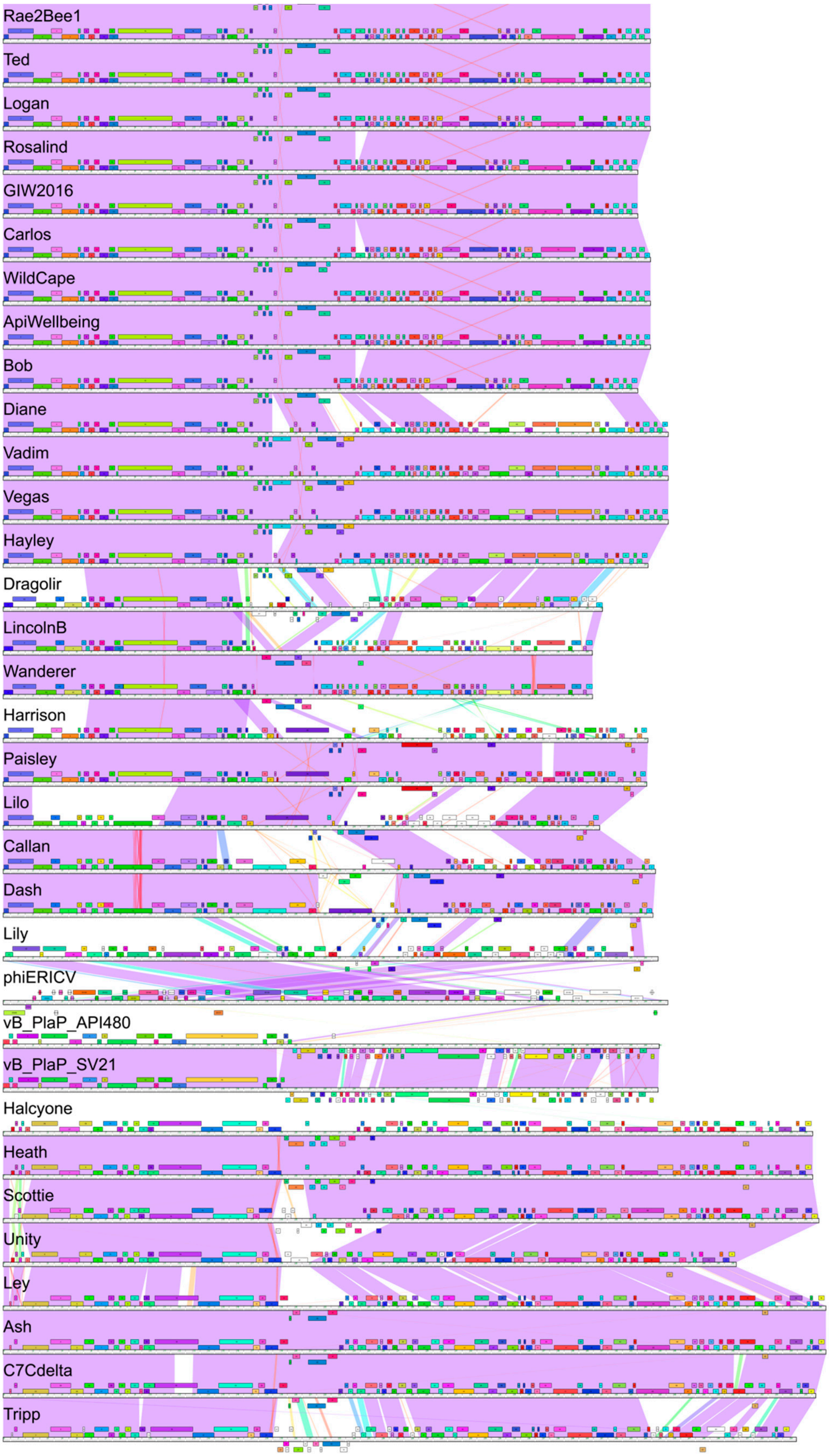

Figure S1. Genome maps of 96 P. larvae phages displayed using Phamerator

Coloured boxes represent genes and genes of the same colour indicate they belong to the same pham. Shading between the genomes indicates how similar an aligned region is at the nucleotide level according to the E-value, with purple depicting an E-value of zero, white indicating no recognisable similarity, and red indicating similarity at the cut-off threshold of 1E-4.

Table S1. 96 P. larvae phages isolated from around the world

Please see spreadsheet

Table S2. 17 *P. larvae* bacterial strains

***P. larvae* Strain**	***P. larvae* Genotype**	**GenBank Accession No.**
PFR-Pl-2017	ERIC I	JARDRG000000000
PFR-Pl-2006	ERIC I	JARDAI000000000
Pl-WAI	ERIC I	JARDRH000000000
Pl-TP	ERIC I	JARDRJ000000000
Pl-CHCH	ERIC I	JARDRI000000000
Pl-F1A	ERIC I	JARDRL000000000
Pl-F2B	ERIC I	JARDRM000000000
Pl-P1627	ERIC I	JARDRK000000000
ATCC 9545	ERIC I	CP019687
DSM 7030	ERIC I	CP019651
DSM 25430	ERIC II	CP003355
SAG 10367	ERIC II	CP020557
LMG 16252	ERIC III	CP019655
ATCC 13537	ERIC IV	CP019794
CCM 38	ERIC IV	CP020327
LMG 16247	ERIC IV	CP019659
DSM 106052	ERIC V	CP019717

Table S3. Clustering of all 96 *P. larvae* phages based on percent identity

Please see spreadsheet

Table S4. Repeat sequences found flanking Plx1 toxin gene. Differences between the Left and Right versions of the repeats are shown in bold.

**Repeat Name**	**Bp**	**Repeat Sequence**	**Direction**	**Start in Phage***	**Start in *P. larvae* ERIC I**
**Plx1FHL**	**39**	ACAGAAAGAGCCTTTGAG**T**AAGCACCTCAAG**A**CTCTTTT	F	18,634	602,646
**Plx1FHR**	**39**	ACAGAAAGAGCCTTTGAG**G**AAGCACCTCAAG**G**CTCTTTT	F	22,583	606,593
**Plx1FDL**	**55**	TCTTGACAAAA**G**CTCTTT**G**CAACCATCTCACCCAAAAACCC**T**TG**T**TTTTGCAACC	F	21,490	-
**Plx1FDR**	**55**	TCTTGACAAAA**A**CTCTTT**T**CAACCATCTCACCCAAAAACCC**G**TG**A**TTTTGCAACC	F	25,425	606,481

*Phage position is specific to the named phage D = Dash and H = Harrison.

Table S5. Spacer sequences found in eight NZ *P. larvae* bacterial strains

	Spacer Sequence	Spacer Length (bp)	Strains containing Spacer	Phages containing Protospacer	Gene containing Protospacer
1	TATGACCGGGTATCTGCTGTTGCAGAAGCCGAAGGGT	37	PFR-Pl-2017<br>PFR-Pl-2006<br>Pl-WAI<br>Pl-TP<br>Pl-CHCH<br>Pl-P1627<br>Pl-F1A<br>Pl-F2B	-	
2	ACGGTATGAATCGTAAGTCGCCTGACTCACGATATG	36	PFR-Pl-2017<br>PFR-Pl-2006<br>Pl-WAI<br>Pl-TP<br>Pl-CHCH<br>Pl-P1627<br>Pl-F1A<br>Pl-F2B	-	

3	CGTTCTGTGGCCAGTGTCATATGGCGACAGGCGAAT	36	PFR-Pl-2017<br>PFR-Pl-2006<br>Pl-TP<br>Pl-CHCH<br>Pl-F1A<br>Pl-F2B	-	
4	AAAAACCCCGATTATGTGGCGGCCGTCCGCACGC	34	PFR-Pl-2017<br>PFR-Pl-2006<br>Pl-WAI<br>Pl-TP<br>Pl-CHCH<br>Pl-P1627<br>Pl-F1A<br>Pl-F2B	-	
5	ACATCATCTGAAACACCCTCAGGAAACAATTCCT	34	PFR-Pl-2017<br>PFR-Pl-2006<br>Pl-WAI<br>Pl-TP<br>Pl-CHCH<br>Pl-F1A<br>Pl-F2B	-	

6	AACAATTACAAATATGCAACTGAAGCAGATGTAAAT	36	PFR-Pl-2017<br>PFR-Pl-2006<br>Pl-WAI<br>Pl-TP<br>Pl-CHCH<br>Pl-F1A<br>Pl-F2B	Callan, Dash<br>100% match	ERF superfamily
7	CATCATCAAAATACAAACCGAATTGATTGCTATTCCC	37	PFR-Pl-2017<br>PFR-Pl-2006<br>Pl-WAI<br>Pl-TP<br>Pl-CHCH<br>Pl-F1A<br>Pl-F2B	-	
8	ACCATGGAAGCGTTGAGACATGGGCCAGAAGATCCA	36	PFR-Pl-2017<br>PFR-Pl-2006<br>Pl-WAI<br>Pl-TP<br>Pl-CHCH<br>Pl-P1627<br>Pl-F1A<br>Pl-F2B	-	

9	GGATAATTTCCGAAAGGTTATTTTGGTTTTCAAT	34	PFR-Pl-2017<br>PFR-Pl-2006<br>Pl-TP<br>Pl-CHCH<br>Pl-P1627<br>Pl-F1A<br>Pl-F2B	-	
10	CAATTAAGCCGACCGCCATATAGCGCGGCTAT	32	PFR-Pl-2017<br>Pl-WAI<br>Pl-TP<br>Pl-CHCH<br>Pl-P1627<br>Pl-F1A<br>Pl-F2B	Callan, Dash, Lilo<br>81.3-84.4% match	Intergenic
11	CCAATTCTTTTTGTTCACAAAGTGCTTTCATCTCC	35	PFR-Pl-2017<br>PFR-Pl-2006<br>Pl-WAI<br>Pl-TP<br>Pl-CHCH<br>Pl-P1627<br>Pl-F1A<br>Pl-F2B	-	

12	GCCAAAGGGCGGATAATTTGAGGAGGGCTGTGTGA	35	PFR-Pl-2017<br>PFR-Pl-2006<br>Pl-WAI<br>Pl-TP<br>Pl-CHCH<br>Pl-P1627<br>Pl-F1A<br>Pl-F2B	-	
13	CTACTTATAACTGCTGAATACACTGTCGCTACTGC	35	PFR-Pl-2017<br>PFR-Pl-2006<br>Pl-WAI<br>Pl-TP<br>Pl-CHCH<br>Pl-P1627<br>Pl-F1A<br>Pl-F2B	-	
14	AAGGGCAGGTACGACGCCCACGCGGCGGGCAAT	33	PFR-Pl-2017<br>PFR-Pl-2006<br>Pl-WAI<br>Pl-TP<br>Pl-CHCH<br>Pl-P1627<br>Pl-F1A<br>Pl-F2B	-	

15	ATACGGAAACGGAAGAGTATTGGGAAATGGAAGAACT	37	PFR-Pl-2017<br>PFR-Pl-2006<br>Pl-WAI<br>Pl-TP<br>Pl-CHCH<br>Pl-P1627<br>Pl-F1A<br>Pl-F2B	-	
16	GGAATGATTCGGTATTCGTCTGCCTGATACTTTTCC	36	PFR-Pl-2017<br>PFR-Pl-2006<br>Pl-WAI<br>Pl-TP<br>Pl-CHCH<br>Pl-P1627<br>Pl-F1A<br>Pl-F2B	-	
17	GGCAAATGGTGGACGGACACGGAATCACCCACACCA	36	PFR-Pl-2017<br>PFR-Pl-2006<br>Pl-WAI<br>Pl-TP<br>Pl-CHCH<br>Pl-P1627<br>Pl-F1A<br>Pl-F2B	All NZ Vegas Cluster<br>91.7% match	DNA Polymerase

18	CATGAAGTAATTGCAGTTTCGAACAACGCTAAAG	34	Pl-P1627	-	
19	ATTGATCGTTTCTTTAAAGACTGCCCAGGCAAA	33	Pl-P1627	-	
20	AAAACACAGTTAAATGGTTTACGCACATAATTTTTTA	37	Pl-P1627	-	
21	ACTAGAGCAATGAGCATTAACGGGATTCCAATCA	34	Pl-P1627	-	
22	CACATCATATGAAGGTGTATGGGAGTGTGAAACA	34	Pl-P1627	-	
23	CCTGAGTGAGTGAAATCAAAGGAGCGATACTGG	33	Pl-P1627	-	
24	GGATGTTAAAAAAGCAACCTTTGACATGGATAT	33	Pl-P1627	-	
25	TCAACGGACGCAAGGTCTATATTGTTTACGACAA	34	Pl-P1627	-	
26	CAATTGCGTTCGGGTTGTGCTTTATACAGTAATT	34	Pl-P1627	-	
27	TGCATACATGTTCTTTTTGCGGCTCCATTGTAACA	35	Pl-P1627	-	
28	GGCTTGGGGCCCTTGTCGCTGCACCGATCAATGCTG	36	Pl-P1627	-	
29	TACTTGGAGTACAGTGGCGTACAAAGTCTACCAGGA	36	Pl-P1627	-	

Table S6. Protospacer sequences found in 17 *P. larvae* bacterial strains and 26 New Zealand phages

	**Strain**	**ERIC Group**	**No. NZ phage found in**	**NZ phage found in**	**Other phage found in**	
[1] "TTACAGGGGCAGGGAGGTACAGAAGATAGGAGGTAC"	DSM25430	ERIC II	3	Callan, Dash, Lilo	Harrison/Paisley	
[2] "GATTTATGGGCACCTATATACCTCCTGGATCAGGGA"	SAG 10367	ERIC II	23	Vegas Cluster	None	
[3] "GTTAGACGAGCGTGTGAGGAGGCTGCAACAGGCA"	SAG 10367	ERIC II	3	Callan, Dash, Lilo	Harrison/Paisley	
[4] "AGGAAACCGCCGTTTCTGGCCCGTATCAGTCAAGCCT"	SAG 10367	ERIC II	23	Vegas Cluster	None	
[5] "GAGCAAGCTGCAACAGAACCGAAATGGACCACT"	DSM 106052	ERIC V	23	Vegas Cluster	LincolnB/Wanderer	
[6] "AACAATTACAAATATGCAACTGAAGCAGATGTAAAT"	DSM 7030	ERIC I	2	Callan, Dash	Harrison/Paisley	Same as protospacer found in NZ bacterial strains
